# Self-perception of voice and knowledge of vocal health and hygiene in *Candomblé* religious leaders in Brazil

**DOI:** 10.1590/2317-1782/20242023087en

**Published:** 2024-08-02

**Authors:** Kenya Ayo-Kianga da Silva Faustino, Felipe Moreti, Mara Behlau

**Affiliations:** 1 Centro de Estudos da Voz - CEV - São Paulo (SP), Brasil.; 2 Departamento de Fonoaudiologia, Faculdade de Filosofia e Ciências, Universidade Estadual Paulista “Júlio de Mesquita Filho” - Unesp - Marília (SP), Brasil.

**Keywords:** Voice, Religious Personnel, Singing, Self-Testing, Surveys and Questionnaires, Knowledge, Signs and Symptoms, Speech, Language and Hearing Sciences

## Abstract

**Purpose:**

To verify possible complaints, voice and aerodigestive symptoms, singing voice handicap, and knowledge of vocal health and hygiene in *Candomblé* religious leaders in Brazil.

**Methods:**

The study comprised 112 individuals who filled out a questionnaire with their identification and characterization, the stratified classification of their professional activities, and their self-perception of voice. Three self-assessment protocols - VoiSS, QSHV, and MSHI - were also used.

**Results:**

The self-assessment of voice ranged from average to good. VoiSS mean total score was 23.04, which is above the cutoff. QSHV mean score was 23.54 points, which is near the cutoff. MSHI mean score (the perception of singing voice handicap) was 25.66 points. There was a substantially strong positive correlation between VoiSS and MSHI total scores (0.789; p<0.001). Women had higher limitation scores (p=0.012) and total scores (p=0.012) in VoiSS and higher handicap scores (p=0.038) in MSHI. Level I professionals - vocal elite (singers and actors) - had significantly higher QSHV scores than those in levels IV (p=0.010) and V (p=0.008). Most respondents had not visited an otorhinolaryngologist (89.29%) within the last year and had not been submitted to speech therapy (83.04%) for voice complaints.

**Conclusion:**

*Candomblé* leaders, particularly women, perceived voice symptoms and singing voice handicaps, with no relationship with their knowledge of vocal health and hygiene. Despite the complaints, most subjects reported not having visited health professionals responsible for voice care within the last year.

## INTRODUCTION

Occupational voice use in religious practices demands flexibility and vocal health to respond to the needs of oral production, expressiveness, and vocal and body adjustments. This use has an emotional value of welcome, advice, and guidance that further increases the worth of the vocal quality. In the case of religious singers, there may also be aspects of religiosity that interfere with vocal behavior, often leading them to sing at high vocal intensity^(1,2)^.

The literature points to high vocal demand, little vocal rest, allergies, and laryngopharyngeal reflux as risk factors in religious people. Furthermore, a high prevalence of complaints such as throat clearing, hoarseness, and laryngeal pain and irritation have been identified^(3,4)^ as compromising vocal comfort and emission quality.

Some religious groups have received attention from researchers regarding the vocal aspects involved in conveying messages, such as evangelical Methodists, Adventists, Presbyterians, Catholics, and Jews ^(1-8)^. However, voice use by leaders of African-based religions, more specifically *Candomblé*, has not yet been subject to scientific analysis. Multiple complex factors probably explain this lack of scientific data, possibly related to the historical processes of invisibility experienced by African-based religions^(9)^.

It can be said that there is a corresponding song for every daily routine gesture or religious practice among *Candomblé* followers. In this context, the sung word is considered the vehicle of vital transmissible energy (*axé*). In public ceremonies, singing is present most of the time, in preparation for what is going to happen, the arrival, the various dances, acts, the return of the African gods (*Orixás*), and at the end, in gratitude for the shared blessings (*ase*)^(10-12)^.

Singing is common to the general followers of the *Candomblé* culture/religion and is practiced by their leaders^(13)^.

The vocal professional responsibilities of leadership in *Candomblé* include singing in most activities of daily living, such as sleeping, waking up, before and after meals, entering and leaving home or work, and planting, harvesting, and preparing herbs and foods. In addition to everyday routines, they sing for rites of passage, care processes, oracular consultations, teaching moments, spiritual counseling, and other internal rituals. It is clear, therefore, that the voice is active in all their activities^(12)^.

Given their great vocal demand, it is important to look at the quality of their voice. The quality of a person's voice interferes with how a message or feeling is socially and professionally conveyed, especially for professionals who use it as the main tool of their trade^(14)^. The perception of the impact of vocal changes can vary greatly among different occupational voice users^(15)^.

The literature on the care of occupational voice users points out the high relevance of knowing about vocal health and hygiene. A search in the literature shows that this knowledge is present in other religious groups^(6)^. However, little has been found about the relationship between this knowledge and vocal self-perception^(16)^.

The investigation of vocal self-perception and aerodigestive symptoms has reported complaints of vocal fatigue and hoarseness after occupational voice use among religious groups. It has also reported a strong relationship between knowledge about vocal care and better voice self-assessment and between self-assessment and the perception of voice problems^(7,17)^.

Considering the need to characterize and reflect on the vocal use of religious leaders of *Candomblé* in Brazil in both speech and singing, this study aimed to verify the self-perception of vocal and aerodigestive symptoms, singing voice handicap, and the knowledge of vocal health and hygiene among *Candomblé* leaders in Brazil.

## METHODS

This prospective, descriptive, cross-sectional study included religious leaders from Brazilian *Candomblé*, approved by the Research Ethics Committee of the *Universidade de Taubaté* (UNITAU), under evaluation report no. 4.541.213, of February 15, 2021.

The sample calculation indicated the inclusion of a minimum N of 97 subjects.

They were initially recruited through direct contact with known religious leaders. Then, two strategies were outlined: one was the snowball method, and the other was the dissemination of the questionnaire link on social media profiles and WhatsApp groups from February 18, 2021, to June 9, 2021. The inclusion criteria were being a leader in *Candomblé* and singing in religious rites.

There was a total of 127 forms. Of these, eight participants were excluded because they reported not singing in religious rites, and seven subjects were excluded because they did not hold leadership positions in *Candomblé*. Hence, the final study sample comprised 112 individuals, who completed the instruments online on Google Forms, initially selecting the “participate in the research” option below the informed consent form, thus authorizing and consenting to their participation and disclosure of data without identifying participants.

All participants answered an identification questionnaire with personal data (name, date of birth, self-declared race/color, sex, occupation, contact), religious data (religious position, time of initiation, nation/strand in *Candomblé*, and whether the person sings in religious rites), and vocal health data (whether they had consulted an otorhinolaryngologist for voice problems in the previous year and whether they had already undergone any Speech-Language Pathology treatment).

A current vocal self-assessment was then requested (excellent, very good, good, fair, or poor voice), and the following vocal self-assessment protocols were applied: the validated Brazilian Portuguese version of the Voice Symptom Scale (VoiSS)^(18)^, a psychometrically robust protocol for surveying the presence and frequency of vocal and aerodigestive symptoms in this population, providing information on functioning, emotional impact, and physical symptoms that a voice problem can cause in an individual's life; the *Questionário de Saúde e Higiene Vocal* (QSHV) (Vocal Health and Hygiene Questionnaire, free translation)^(19)^, developed and validated in Brazilian Portuguese, to verify the *Candomblé* leaders’ knowledge about health and vocal habits; and the translated and culturally adapted version into Brazilian Portuguese of the Modern Singing Handicap Index (MSHI)^(15)^ to check whether this population has any singing voice handicap, given their vocal health conditions.

The professions were analyzed with the classification proposed by Koufman and Isacson^(20)^, which categorizes occupational voice use according to demand and the impact of a possible voice problem on the continuity of their occupation. Thus, level I encompasses the vocal elite, professionals whose careers can be seriously affected due to a vocal disorder, such as singers and actors; level II: speaking voice professionals, for whom a moderate vocal change could cause a professional impact, as occurs with most teachers, lawyers, Speech-Language Pathologists, telemarketers, and so forth; level III: non-occupational voice users, whose professional activities would be impaired only by severe dysphonia, such as physicians and salespeople; level IV: non-occupational non-voice users, who are not limited even with extreme vocal impairment, such as designers, computer programmers, clerks, and so on. This study included level V, i.e., unemployed participants, retirees, and students, who do not fall into the previous classifications, as also used in other studies^(21,22)^.

VoiSS identifies vocal and aerodigestive symptoms. Each question score ranges from 0 to 4, with 0 being never, 1 occasionally, 2 some of the time, 3 most of the time, and 4 always. The total VoiSS score indicates the general level of voice change (maximum 120), with impairment (maximum 60), emotional (maximum 32), and physical (maximum 28) subscales. The cutoff of the total score is 16 points^(18,23)^.

The QSHV was developed and validated in Brazilian Portuguese and provides information on knowledge about vocal health and hygiene. Each question is marked according to the individual's beliefs, and the answers are divided into positive, neutral, and negative items, with one point being scored for each correct answer according to the questionnaire template, whose cutoff is 23 points^(19)^.

The MSHI has 30 items to explore the singing handicap perception, distributed into three subscales - disability, handicap, and impairment, which correspond respectively to the functional, emotional, and organic domains. Responses are scored according to frequency of occurrence: 0: never, 1: almost never, 2: sometimes, 3: almost always, and 4: always. Each subscale has a maximum value of 40 points, and the total (the sum of the previous ones) has a maximum score of 120 points. The higher the score, the higher the singing voice handicap perceived by the individual. This protocol was originally developed in Italian and was translated and culturally adapted to Brazilian Portuguese^(15)^.

### Data analysis

The data were analyzed descriptively and inferentially using SPSS 25.0 software. The significance level was set at 5% for inference analyses.

The descriptive analysis of continuous quantitative and ordinal qualitative variables calculated the measures of central tendency (mean and median), variability (standard deviation), and position (minimum, maximum, and quartiles one and three). The descriptive analysis of nominal qualitative variables calculated the absolute frequency and relative percentage frequency.

Quantitative variables were analyzed using the Shapiro-Wilk test, and all had non-normal distribution. The inferential analysis comparing ordinal quantitative and qualitative variables as a function of two independent groups was performed using the Mann-Whitney test. The inferential analysis comparing ordinal quantitative and qualitative variables as a function of multiple independent groups was performed using the Kruskal-Wallis test. Significant values were subjected to pairwise analysis. The correlation between ordinal qualitative and quantitative variables was performed using the Spearman correlation test. The Landis and Koch Kappa coefficient was used to analyze the strength of correlations^(24)^.

## RESULTS

The study comprised 112 *Candomblé* religious leaders in Brazil, with a mean age of 42.63 years, ranging from 17 to 67 years, and with a mean time of initiation into the religion of 18.93 years, ranging from less than a year to 52 years. The descriptive analysis of sample characterization can be found in Table 1.

**Table 1:** Descriptive analysis of nominal qualitative variables characterizing the sample of *Candomblé* leaders in Brazil

Variable and categories	N	%
Race/color		
White	20	17.86
Black	70	62.50
Multiracial	21	18.75
Others	1	0.89
Sex		
Males	56	50.00
Females	56	50.00
Profession categorization		
I	9	8.04
II	30	26.79
III	22	19.64
IV	39	34.82
V	12	10.71
Religious function in *Candomblé*		
*Ogan*	20	17.86
Father leadership	28	25.00
Mother leadership	35	31.25
*Egbomi*	15	13.39
*Ekede*	14	12.50
*Candomblé* nation		
*Ketu*	85	75.89
*Jeje*	8	7.14
*Angola*	7	6.25
*Nago Ebga*	3	2.68
*Efon*	2	1.79
*Yoruba*	3	2.68
Others	4	3.57
Sings in religious rites		
Yes	112	100.00
Visited an otorhinolaryngologist for voice problems in the previous year/reason		
No	100	89.29
Sore throat	6	5.36
Hoarseness	3	2.68
Voicelessness	1	0.89
Others	2	1.79
Speech-Language Pathology therapy		
No	93	83.04
Yes	15	13.39
Others	4	3.57

Descriptive analysis

**Caption:** N = absolute frequency; % = relative frequency

As seen in Table 1, most subjects in the distribution of race/color declared themselves Black (62.50%). There was also an equal distribution between sexes (50%). The distribution of profession categorization identified more religious leaders who exercise level IV professions (34.82%), followed by levels II (26.79%) and III (19.64%); levels V (10.71%) and I (8.04%) had the fewest subjects. Another aspect that draws attention is that 89.29% of the subjects had not visited an otorhinolaryngologist in the previous year, and 83.04% never had Speech-Language Pathology therapy.

Table 2 describes the variables of current self-perception of voice and vocal symptoms, knowledge of vocal health and hygiene, and self-perception of modern singing voice handicaps. Regarding the current vocal self-assessment, the mean report was from a good to fair voice. The mean total VoiSS score was 23.04 points, above the 16-point cutoff of the protocol validated for Brazilian Portuguese that separates dysphonic individuals from those who are vocally healthy^(18,23)^. The mean QSHV total score was 23.54 points, within the normal range of the instrument's cutoff for healthy individuals (≥ 23 points)^(19)^. The mean MSHI total score was 25.66; the Disability score was 8.80 points; the Handicap score was 4.92 points; and the Impairment score was 11.94 points - as indicated in Table 2.

**Table 2:** Descriptive analysis of quantitative self-assessment variables in *Candomblé* leaders in Brazil

Variables	Mean	SD	Minimum	Maximum	Q_1_	Median	Q_3_
Current self-perception of voice	2.22	0.97	0.00	4.00	2.00	2.00	3.00
VoiSS							
Impairment	13.15	10.86	0.00	55.00	5.00	10.00	18.00
Emotional	3.20	5.55	0.00	24.00	0.00	0.00	4.00
Physical	6.70	5.58	0.00	25.00	3.00	5.50	9.00
Total	23.04	20.21	0.00	99.00	10.00	16.00	28.75
QSHV Total	23.54	6.76	5.00	31.00	20.00	26.00	29.00
MSHI							
Disability	8.80	8.10	0.00	40.00	2.00	6.50	13.00
Handicap	4.92	6.88	0.00	40.00	0.00	2.00	7.00
Impairment	11.94	10.01	0.00	40.00	3.00	10.00	19.00
Total	25.66	22.71	0.00	119.00	6.00	22.00	35.00

Descriptive analysis

**Caption:** SD = standard deviation; Q_1_ = quartile one; Q_3_ = quartile three; VoiSS = Voice Symptom Scale; QSHV = *Questionário de Saúde e Higiene Vocal*; MSHI = Modern Singing Handicap Index

The current vocal self-assessment was positively correlated^(24)^ with all self-perception domains of vocal symptoms and voice handicaps in modern singing among *Candomblé* religious leaders in Brazil. Only knowledge about vocal health and hygiene was not correlated^(24)^ with the current vocal self-assessment variables.

The self-perception of the voice was moderately positively correlated^(24)^ with the VoiSS Impairment, Emotional, and Total scores; MSHI Disability, Impairment, and Total scores; and weakly correlated with the VoiSS Physical score and MSHI Handicap score. The VoiSS Impairment score was almost perfectly positively correlated with^(24)^ with VoiSS total score and substantially positively correlated^(24)^ with VoiSS Emotional and Physical scores, and MSHI Disability, Handicap, Impairment, and Total scores. The VoiSS Emotional score was substantially positively correlated^(24)^ with the VoiSS total score and MSHI Handicap score and moderately positively correlated^(24)^ with the VoiSS Physical score and MSHI Disability, Impairment, and Total scores.

The VoiSS Physical score was almost perfectly positively correlated^(24)^ with the VoiSS total score, substantially positively correlated^(24)^ with MSHI Impairment and Total scores, and moderately positively correlated^(24)^ with MSHI Disability and Handicap.

The VoiSS Total score was substantially positively correlated^(24)^ with MSHI Disability, Handicap, Impairment, and Total scores. The MSHI Disability score was almost perfectly positively correlated^(24)^ with the MSHI Total score and substantially correlated^(24)^ with the MSHI Disability and Impairment scores. The MSHI Handicap score was substantially positively correlated^(24)^ with the MSHI Total score and moderately correlated^(24)^ with the MSHI Impairment score. MSHI Impairment was almost perfectly positively correlated^(24)^ with MSHI Total score, as seen in Table 3.

**Table 3:** Correlation between self-assessment quantitative variables in *Candomblé* leaders in Brazil

		VoiSS Impairment	VoiSS Emotional	VoiSS Physical	VoiSS Total	QSHV Total	MSHI Disability	MSHI Handicap	MSHI Impairment	MSHI Total
Current self-perception of voice	R	0.505	0.463	0.362	0.517	-0.025	0.485	0.305	0.459	0.488
	p-value	<0.001*	<0.001*	<0.001*	<0.001*	0.791	<0.001*	<0.001*	<0.001*	<0.001*
VoiSS Impairment	R		0.664	0.720	0.964	0.043	0.682	0.621	0.766	0.767
	p-value		<0.001*	<0.001*	<0.001*	0.650	<0.001*	<0.001*	<0.001*	<0.001*
VoiSS Emotional	R			0.511	0.736	0.038	0.577	0.628	0.504	0.604
	p-value			<0.001*	<0.001*	0.694	<0.001*	<0.001*	<0.001*	<0.001*
VoiSS Physical	R				0.834	0.107	0.544	0.477	0.643	0.613
	p-value				<0.001*	0.260	<0.001*	<0.001*	<0.001*	<0.001*
VoiSS Total	R					0.072	0.711	0.641	0.775	0.789
	p-value					0.452	<0.001*	<0.001*	<0.001*	<0.001*
QSHV Total	R						0.013	-0.029	0.087	0.054
	p-value						0.895	0.764	0.362	0.575
MSHI Disability	R							0.662	0.794	0.937
	p-value							<0.001*	<0.001*	<0.001*
MSHI Handicap	R								0.605	0.752
	p-value								<0.001*	<0.001*
MSHI Impairment	R									0.929
	p-value									<0.001*

* Significant values (p ≤ 0.05) – Spearman correlation test

**Caption:** R = correlation coefficient; VoiSS = Voice Symptom Scale; QSHV = *Questionário de Saúde e Higiene Vocal*; MSHI = Modern Singing Handicap Index

The quantitative self-assessment analysis revealed no difference in religious position and *Candomblé* nation among the *Candomblé* religious leaders in Brazil.

Given the absence of significant statistical difference between positions and nations of the religion, the analysis addressed the larger group of *Candomblé* leaders in Brazil.

Female *Candomblé* leaders in Brazil had higher scores than males in self-perceived vocal symptoms in the Impairment (p = 0.012) and total scores (p = 0.012) and self-perceived modern singing voice handicap in the Handicap domain (p = 0.008), as shown in Table 4.

**Table 4:** Analysis of quantitative self-assessment variables per sex in *Candomblé* leaders in Brazil

Variable	Sex	Mean	SD	Minimum	Maximum	Q_1_	Median	Q_3_	p-value
Current self-perception of voice	Males	2.13	0.95	0.00	4.00	2.00	2.00	3.00	0.330
	Females	2.32	0.99	0.00	4.00	2.00	2.00	3.00	
VoiSS									
Impairment	Males	11.09	10.30	0.00	45.00	4.00	8.50	14.00	0.012*
	Females	15.21	11.10	1.00	55.00	7.00	13.00	19.75	
Emotional	Males	2.91	5.74	0.00	24.00	0.00	0.00	2.75	0.137
	Females	3.48	5.38	0.00	22.00	0.00	1.00	4.75	
Physical	Males	5.73	5.22	0.00	21.00	1.25	5.00	8.00	0.052
	Females	7.66	5.81	0.00	25.00	3.25	6.00	10.75	
Total	Males	19.73	19.80	0.00	90.00	7.00	12.00	26.00	0.012*
	Females	26.36	20.24	2.00	99.00	12.25	22.50	31.50	
QSHV Total	Males	23.82	6.46	9.00	31.00	19.50	26.00	29.00	0.659
	Females	23.25	7.10	5.00	30.00	20.00	26.00	29.00	
MSHI									
Disability	Males	8.02	7.65	0.00	28.00	2.00	5.50	12.75	0.233
	Females	9.59	8.53	0.00	40.00	4.00	7.00	13.00	
Handicap	Males	4.21	6.43	0.00	26.00	0.00	1.00	7.00	0.038*
	Females	5.63	7.30	0.00	40.00	1.00	3.00	7.00	
Impairment	Males	10.18	9.61	0.00	40.00	2.00	8.50	17.00	0.053
	Females	13.70	10.17	0.00	39.00	6.25	12.50	20.00	
Total	Males	22.41	21.47	0.00	89.00	5.00	16.00	31.75	0.093
	Females	28.91	23.62	0.00	119.00	13.00	25.50	40.00	

* Significant values (p ≤ 0.05) - Mann-Whitney test

**Caption:** SD = standard deviation; Q_1_ = quartile one; Q_3_ = quartile three; VoiSS = Voice Symptom Scale; QSHV = *Questionário de Saúde e Higiene Vocal*; MSHI = Modern Singing Handicap Index

The quantitative self-assessment variables were not correlated with age and length of initiation in *Candomblé* leaders in Brazil. There was a difference in knowledge of vocal health and hygiene in relation to the profession categorization among *Candomblé* leaders in Brazil (p = 0.008). Pairwise analysis showed that level I professionals had significantly higher scores than level IV (p = 0.010) and level V professionals (p = 0.008), as shown in Table 5.

**Table 5:** Analysis of quantitative self-assessment variables based on profession categorization in *Candomblé* leaders in Brazil

Variable	Profession categorization	Mean	SD	Minimum	Maximum	Q_1_	Median	Q_3_	p-value	Pairwise
Current self-perception of voice	I	2.22	1.39	0.00	4.00	1.00	2.00	3.50	0.860	
	II	2.33	0.88	0.00	4.00	2.00	2.00	3.00		
	III	2.09	0.81	1.00	4.00	1.75	2.00	3.00		
	IV	2.26	0.99	0.00	4.00	2.00	2.00	3.00		
	V	2.08	1.16	0.00	4.00	1.00	2.00	3.00		
VoiSS										
Impairment	I	12.78	10.63	2.00	36.00	5.50	9.00	18.50	0.874	
	II	11.77	8.08	1.00	32.00	5.00	11.00	16.50		
	III	11.64	8.74	0.00	35.00	4.00	11.00	17.25		
	IV	15.74	13.68	0.00	55.00	4.00	10.00	23.00		
	V	11.25	10.25	2.00	34.00	5.00	7.00	12.75		
Emotional	I	5.11	6.43	0.00	19.00	0.50	2.00	9.00	0.410	
	II	2.53	4.76	0.00	22.00	0.00	0.00	3.25		
	III	3.09	4.40	0.00	16.00	0.00	0.50	6.00		
	IV	3.56	6.47	0.00	24.00	0.00	0.00	4.00		
	V	2.42	5.81	0.00	20.00	0.00	0.00	1.75		
Physical	I	6.78	7.50	1.00	25.00	2.00	3.00	9.00	0.554	
	II	6.53	3.79	0.00	13.00	4.00	6.00	10.00		
	III	5.82	5.07	0.00	20.00	1.00	5.00	8.25		
	IV	7.90	6.79	0.00	22.00	2.00	6.00	13.00		
	V	4.75	4.05	0.00	15.00	2.25	3.50	6.00		
Total	I	24.67	24.17	5.00	80.00	8.50	13.00	36.50	0.870	
	II	20.83	14.00	1.00	51.00	10.75	16.00	28.50		
	III	20.55	16.10	0.00	71.00	8.50	19.50	26.25		
	IV	27.21	25.59	0.00	99.00	10.00	16.00	42.00		
	V	18.42	17.57	4.00	63.00	6.75	12.00	25.25		
QSHV total	I	28.78	1.92	24.00	30.00	28.50	29.00	30.00	0.008*	I > IV (p=0.010) = V (p=0.008)
	II	24.57	6.22	8.00	31.00	24.50	26.50	29.00		
	III	24.27	4.91	12.00	30.00	21.25	25.50	28.00		
	IV	22.31	7.42	5.00	31.00	16.00	26.00	28.00		
	V	19.67	8.54	6.00	29.00	10.75	20.50	29.00		
MSHI										
Disability	I	7.78	7.31	1.00	19.00	1.00	6.00	15.00	0.946	
	II	7.97	6.96	0.00	23.00	3.75	5.50	11.25		
	III	8.00	7.42	0.00	2.00	3.00	0.00	0.00		
	IV	9.97	9.32	0.00	40.00	2.00	7.00	15.00		
	V	9.33	9.05	0.00	28.00	1.50	6.50	16.75		
Handicap	I	6.11	6.62	0.00	17.00	0.50	3.00	13.00	0.847	
	II	4.03	6.18	0.00	26.00	0.00	2.00	4.00		
	III	3.82	4.54	0.00	16.00	0.00	2.50	7.00		
	IV	6.08	8.25	0.00	40.00	0.00	3.00	8.00		
	V	4.50	7.78	0.00	28.00	0.25	2.00	6.00		
Impairment	I	11.67	8.77	1.00	24.00	3.00	10.00	20.00	0.953	
	II	12.37	9.12	0.00	40.00	6.50	11.00	19.25		
	III	10.45	9.01	0.00	37.00	2.50	10.50	14.25		
	IV	12.23	10.65	0.00	39.00	2.00	11.00	20.00		
	V	12.83	13.50	0.00	36.00	1.25	8.00	26.00		
Total	I	25.56	22.07	3.00	59.00	5.00	26.00	48.50	0.964	
	II	24.37	20.24	0.00	589.00	12.50	21.00	33.00		
	III	22.27	19.49	0.00	82.00	5.50	21.50	32.25		
	IV	28.28	25.83	0.00	119.00	6.00	24.00	45.00		
	V	26.67	26.17	1.00	90.00	5.75	18.50	44.75		

* Significant values (p ≤ 0.05) - Kruskal-Wallis test

**Caption:** SD = standard deviation; Q_1_ = quartile one; Q_3_ = quartile three; VoiSS = Voice Symptom Scale; QSHV = *Questionário de Saúde e Higiene Vocal*; MSHI = Modern Singing Handicap Index

## DISCUSSION

*Candomblé* people face numerous vocal challenges, as the intensive voice use in the religious context is often accompanied by other professions that likewise depend on the voice. Nevertheless, they seldom seek vocal healthcare, prevention, and rehabilitation/treatment services, such as otorhinolaryngologists and Speech-Language Pathologists (Table 1).

Most vocal self-assessment responses in this study are between good and fair voices, which may explain the low demand for specialized professionals. Also, as among popular singers^(16)^, small vocal changes may be part of the vocal signature and, in some cases, are not reasons for seeking health professionals.

Vocal self-perception analysis is a strategy commonly used in voice and self-assessment protocols research, including Brazilian studies to validate voice self-assessment protocols^(25)^. It has proven to be an important measure for the vocal diagnosis of patients with dysphonia^(26)^ since voice problems can compromise quality of life.

The results indicate that this population has high scores for vocal and aerodigestive symptoms and the perception of the modern singing impact and handicap in the disability, handicap, and impairment scores.

The mean VoiSS total score in the sample was 23.04 (Table 2), approximately seven points above the cutoff for the total score that separates dysphonic individuals from vocally healthy individuals (16 points)^(18,23)^ - which shows that *Candomblé* leaders in this study have and perceive signs and symptoms of vocal and aerodigestive changes. Nonetheless, this value is lower than the mean found among amateur Baptist soloists (30.9 points)^(27)^. Vocal health complaints were also found in most evangelical leaders^(5,7)^.

The mean QSHV scores (Table 2) are close to the cutoff, which distinguishes vocal health knowledge between vocally healthy and dysphonic individuals. This result, however, is below the mean scores achieved in the literature by vocally healthy individuals, which is 29.12 points^(19)^ and different from those of evangelical pastors^(6)^, as most subjects had good knowledge about voice health and hygiene, with mean scores of 28 points.

The descriptive analysis also found a mean MSHI total score of 25.66 (Table 2). Although the literature does not indicate a cutoff for Brazilian Portuguese in this protocol, this value was close to those found in other Brazilian studies, such as Moreti et al.^(15)^, in amateur choir singers with vocal complaints, with 26.91 points. Also, in Sales et al.^(28)^, the mean total score for popular singers was 16.6 points, indicating less handicap than in the population in this study.

This study found a positive correlation between current vocal self-assessment and all self-perception domains of vocal symptoms and modern singing voice handicap among *Candomblé* leaders in Brazil, which may show that the more the individual perceives their voice changes, the lower their vocal performance (Table 3). A vocal problem can reflect on how the subject evaluates their voice and, although not all dysphonia manifests itself as a change in vocal quality, the literature points to a positive correlation between the self-perception of vocal problems and its negative impact on quality of life^(25,26)^.

There was a statistically significant correlation between knowledge about vocal health and hygiene and the current vocal self-assessment (Table 3), a result different from that found by Coelho et al.^(16)^ (in which the perception of vocal symptoms was not related to the degree of knowledge of vocal health and hygiene in the study singers) and Roza et al.^(17)^ (in which individuals with more vocal care knowledge had better voice self-assessment). Thus, like pastors, *Candomblé* leaders can be considered part of a population at vocal risk in the workplace^(6)^.

The positive correlation between vocal self-assessment and VoiSS and MSHI (Table 3) shows that the higher the reported symptom score, the greater the subject's singing handicap, as also demonstrated by Lopes and Ghirard^(27)^.

Only knowledge about vocal health and hygiene did not correlate with the vocal self-assessment variables (Table 3), indicating that knowledge about vocal health may not be enough to guarantee the reduction of vocal and aerodigestive symptoms.

There was no correlation between the quantitative self-assessment variables and the age and length of initiation in *Candomblé* leaders in Brazil (Table 3) - unlike the study by Lobo et al.^(6)^, who found higher QSHV scores in pastors with a longer pastoral career.

Females had significantly higher VoiSS Impairment and total scores for self-perception of vocal symptoms and MSHI Handicap scores for self-perception of vocal handicap in modern singing than males (Table 4).

This relationship of higher scores in females was also found in evangelical female singers and classical and popular male and female singers^(5,8,16)^. For these researchers, the fact that women have a greater voice handicap than men may indicate the ability to perceive more clearly any organic, functional, or emotional restriction in the ability to sing^(8)^ – unlike Souza et al.^(7)^, who a prevalence among males.

Thus, knowledge of vocal health and hygiene may not have been enough to prevent vocal symptoms from being noticed, given that it was not statistically correlated with the other variables.

Regarding the profession categorization among religious leaders (Table 5), the QSHV identified that level I professionals (vocal elite) had significantly higher scores than group IV professionals (retirees and those not limited even in extreme vocal impairments). This may suggest that knowledge of vocal health and hygiene is more related to the professional category than to the religious function. It is important to highlight the relationship between work and vocal disorders, since occupational voice users had a higher frequency of work-related vocal disorders, further demonstrating the increasing relevance of vocal healthcare among occupational voice users, including religious leaders^(29,30)^.

Thus, Speech-Language Pathology therapy in religious institutions can help prevent possible vocal changes, as people have little knowledge about voice use^(1)^.

Data collection with online forms increases the importance of variables such as current vocal self-assessment, which highlights the person’s perception of the quality of their voice.

The protocols used in this research were complementary, as they cover different quantitative and qualitative dimensions of vocal self-perception, knowledge of vocal health and hygiene, and the perception of singing handicaps to address individuals comprehensively.

Based on this study, it can be stated that the knowledge about vocal health and hygiene among *Candomblé* leaders in Brazil reduces the risk of developing voice changes. However, such knowledge needs to be applied in the daily routine, as alone it does not guarantee vocal health^(6)^.

It is important to think that applying voice assessment protocols along with self-perception ones can provide more complete data about this population’s vocal quality.

It is worth mentioning that few studies address singing in African religions such as *Umbanda* and *Candomblé*. This is an important field for Speech-Language Pathology research and practice, given the high scores in the vocal symptoms and aerodigestive and singing handicap protocols. It is also necessary to expand vocal healthcare and hygiene actions, as most leaders tend to practice singing and religious functions for years without ever having studied the subject or using vocal techniques^(8)^.

## CONCLUSION

Brazilian *Candomblé* leaders in this study had signs and symptoms of vocal and aerodigestive changes and singing handicaps. This perception was more evident in women.

The different religious leaders had good knowledge of vocal health regardless of sex. However, this knowledge was not correlated with self-perceived vocal symptoms or singing voice handicaps.

Despite the identification of vocal symptoms, most subjects reported not having visited vocal healthcare professionals in the previous year.

There is a perception that such symptoms influence voice self-assessment. However, knowledge about vocal health alone was not enough to protect this population. It is very important to raise awareness and promote access to vocal health services.
