# Supplementary material for: Self-perception of voice and knowledge of vocal health and hygiene in Candomblé religious leaders in Brazil
Source: Codas. 2024 Aug 2;36(4):e20230087. doi: 10.1590/2317-1782/20242023087en (PMC11340867; doi:10.1590/2317-1782/20242023087en)
Supplement: Translation

# Translation: Autopercepção da voz e conhecimento em saúde e higiene vocal de líderes religiosos do Candomblé no Brasil

**DOI:** 10.1590/2317-1782/20242023087pt

**Authors:** Kenya Ayo-Kianga da Silva Faustino, Felipe Moreti, Mara Behlau

**Affiliations:** 1 Centro de Estudos da Voz - CEV - São Paulo (SP), Brasil.; 2 Departamento de Fonoaudiologia, Faculdade de Filosofia e Ciências, Universidade Estadual Paulista “Júlio de Mesquita Filho” - Unesp - Marília (SP), Brasil.

**Keywords:** Voz, Religiosos, Canto, Autoteste, Inquéritos e Questionários, Conhecimento, Sinais e Sintomas, Fonoaudiologia

## Abstract

**Objetivo:**

Verificar a autopercepção de sintomas vocais e aerodigestivos, desvantagem vocal no canto e o conhecimento em saúde e higiene vocal em líderes religiosos do Candomblé do Brasil.

**Método:**

Participaram deste estudo 112 indivíduos, que preencheram virtualmente um questionário de identificação, caracterização, classificação das atividades profissionais em níveis e autoavaliação vocal no momento atual, além de três protocolos de autoavaliação: Escala de Sinais e Sintomas (ESV), Questionário de Saúde e Higiene Vocal (QSHV) e Índice de Desvantagem para o Canto Moderno (IDCM). Foram utilizados os testes de correlação de Spearman, teste de Mann-Whitney e teste de Kruskal-Wallis.

**Resultados:**

A autoavaliação vocal no momento atual variou de razoável a boa. A média do escore total na ESV foi de 23,04, acima da nota de corte. O QSHV apresentou valor médio de 23,54 pontos, próximo à nota de corte. O Índice de Desvantagem para o Canto Moderno (IDCM) mostrou média de 25,66 pontos. Houve correlação positiva de força substancial entre os escores totais da ESV e IDCM (0,789). Mulheres apresentaram maiores escores no ESV total e ESV limitação, além de maior escore no IDCM desvantagem. Profissionais do nível I - elite vocal (cantores e atores) apresentaram escores significativamente maiores para o QSHV que os profissionais do grupo IV (usuário não profissional não-vocal) e os do nível V (pessoas que estão fora do mercado de trabalho). A maioria dos respondentes não consultou Otorrinolaringologista (89,29%) no último ano e nem realizou fonoterapia (83,04%) por queixas vocais.

**Conclusão:**

Líderes do Candomblé apresentaram percepção de sintomas vocais e desvantagem vocal no canto, mais evidente nas mulheres, não havendo relação com o conhecimento de saúde e higiene vocal. Apesar da identificação de sintomas vocais, a maior parte dos sujeitos relatou não ter acessado profissionais de saúde responsáveis por esses cuidados com a voz no último ano.

## INTRODUÇÃO

Entre os grupos de profissionais da voz, o uso no exercício religioso demanda flexibilidade e saúde vocal para responder às necessidades de produção oral, de expressividade, de ajustes vocais e corporais. Este uso se reveste de um valor emocional de acolhimento, aconselhamento e orientação que faz com que o valor da qualidade vocal em si seja ainda maior. No caso de cantores religiosos, pode haver ainda a presença de aspectos da religiosidade que interferem no comportamento vocal, levando-os, muitas vezes, a praticarem seus cantos em intensidade vocal elevada^(1,2)^.

A literatura aponta como fatores de risco em religiosos a alta demanda vocal, tempo de repouso vocal reduzido, alergias e refluxo laringofaríngeo. Além disso, tem-se identificado grande prevalência de queixas como pigarro, rouquidão e dor e irritação laríngea^(3,4)^, que podem comprometer o conforto vocal, e a qualidade da emissão.

Alguns grupos religiosos têm recebido atenção de pesquisadores, quanto aos aspectos vocais envolvidos na transmissão da mensagem, tais como evangélicos metodistas, adventistas, presbiterianos, católicos, judeus^(1-8)^. Contudo, o uso da voz pelos líderes de religiões de matriz africana, mais especificamente o Candomblé, não foram ainda alvo de análise científica. Fatores que expliquem essa falta de dados científicos são provavelmente múltiplos e complexos, podendo estar relacionados aos próprios processos históricos de invisibilidade vivenciados pelas religiões de matriz africana^(9)^.

Para os adeptos do Candomblé, pode-se dizer que para cada gesto constituinte da rotina diária, ou da prática religiosa, há uma cantiga correspondente. Nesse contexto, a palavra cantada é considerada o veículo da energia vital - axé - energia que se transmite. Nas cerimônias públicas, o canto está presente na maior parte do tempo, na preparação para o que vai ocorrer, para a chegada, para as diversas danças e atos e retorno dos deuses africanos - Orixás e ao final em agradecimento às bênçãos - ase compartilhadas^(10-12)^.

Na cultura/religião candomblecista o canto é comum aos adeptos em geral e é entoado por seus líderes^(13)^.

A atribuição profissional vocal da liderança no Candomblé abrange o canto na maior parte das atividades da vida diária, como dormir, acordar, antes e depois das refeições, entrar e sair de casa ou do trabalho, para plantio, colheita e preparo de ervas e alimentos. Além das rotinas do dia a dia, canta-se para os ritos de passagem, processos de cuidado, consultas oraculares, momentos de ensino, aconselhamentos espirituais, assim como para os demais rituais internos. Percebe-se, portanto, que a voz está ativa em todas as atribuições^(12)^.

Diante da grande demanda vocal desta população, torna-se importante olhar para a qualidade desta voz. A qualidade da voz de um indivíduo interfere na transmissão da mensagem ou do sentimento, social e profissionalmente, principalmente para os profissionais que a utilizam como instrumento principal de sua atividade laboral^(14)^. A percepção do impacto da alteração vocal pode ser muito variável entre os diversos profissionais da voz^(15)^.

Um aspecto de grande relevância na literatura sobre os cuidados dos profissionais da voz é o conhecimento sobre saúde e higiene vocal. Quando buscamos a literatura, encontramos que este conhecimento está presente em outros grupos de religiosos^(6)^. No entanto, pouco se tem encontrado sobre a relação entre esse conhecimento e autopercepção vocal^(16)^.

A investigação da autopercepção vocal e de sintomas aerodigestivos tem evidenciado em grupos de religiosos relato de queixas de cansaço vocal e rouquidão após o uso da voz profissional. Relata-se ainda a forte relação entre conhecimento em cuidados vocais e melhor autoavaliação de voz e sobre a relação entre a autoavaliação e a percepção de problemas de voz^(7,17)^.

Considerando a necessidade de caracterização e a reflexão sobre o uso vocal de líderes religiosos do Candomblé do Brasil, tanto na modalidade de voz falada como na cantada, o objetivo desse trabalho foi verificar a autopercepção de sintomas vocais e aerodigestivos, desvantagem vocal no canto e o conhecimento em saúde e higiene vocal em líderes religiosos do Candomblé do Brasil.

## MÉTODOS

Trata-se de estudo transversal descritivo prospectivo, que incluiu líderes religiosos do Candomblé do Brasil, aprovado pelo Comitê de Ética em Pesquisa da Universidade de Taubaté - UNITAU, sob o parecer nº 4.541.213 de 15 de fevereiro de 2021.

Foi realizado o cálculo amostral, chegando-se ao N mínimo de 97 sujeitos a serem incluídos.

O recrutamento ocorreu inicialmente pelo contato direto com lideranças religiosas conhecidas. Em seguida, foram traçadas duas estratégias: uma se deu por estratégia bola de neve e a outra pela divulgação do link do questionário nos perfis de rede sociais e grupos de WhatsApp no período de 18/02/2021 a 09/06/2021. Como critérios de inclusão, foram considerados a ocupação de cargo de liderança no Candomblé e cantar nos ritos religiosos.

Houve o total de 127 formulários. Destes, oito participantes foram excluídos em função de referirem não cantar nos ritos religiosos e sete sujeitos foram excluídos por não ocuparem cargos de liderança no Candomblé. O número final de indivíduos incluídos no estudo foi 112, que preencheram os instrumentos de forma online por meio da plataforma Google *Forms*, inicialmente selecionando a opção “participar da pesquisa” abaixo do Termo de Consentimento Livre e Esclarecido autorizando e consentindo a sua participação e divulgação dos dados sem a identificação dos participantes.

Todos os participantes preencheram um Questionário de Identificação, contendo dados de identificação pessoal (nome, data de nascimento, autodeclaração de raça/cor, gênero, profissão, contato), identificação religiosa (contendo cargo religioso, tempo de iniciado, nação/vertente no Candomblé e se a pessoa canta nos ritos religiosos) e aspectos relacionados à saúde vocal (se houve consulta a um otorrinolaringologista por problemas de voz no último ano e se já fez algum tratamento terapêutico fonoaudiológico).

Em seguida foi solicitada uma autoavaliação vocal no momento atual (voz excelente, muito boa, boa, razoável ou ruim) e a aplicação dos seguintes protocolos de autoavaliação vocal: a versão validada para o português brasileiro da Escala de Sintomas Vocais - ESV^(18)^ protocolo psicometricamente robusto para o levantamento da presença e frequência de sintomas vocais e aerodigestivos nesta população trazendo informações de funcionalidade, impacto emocional, e sintomas físicos que um problema de voz pode acarretar na vida de um indivíduo; a versão desenvolvida e validada em português brasileiro do Questionário de Saúde e Higiene Vocal - QSHV^(19),^ para verificarmos o conhecimento que as lideranças de Candomblé tem sobre saúde e hábitos vocais e a versão traduzida e culturalmente adaptada para o português brasileiro do Índice de Desvantagem para o Canto Moderno - IDCM^(15)^ para verificarmos se há alguma desvantagem vocal no canto desta população diante das condições de saúde vocal encontradas.

Para a análise das profissões, optou-se pela utilização da classificação proposta por Koufman e Isacson^(20)^ que categorizam o uso profissional da voz de acordo com a demanda e o impacto de um eventual problema de voz para a continuidade da vida laboral. Desta forma, considera-se nível I: elite vocal, os profissionais cujas carreiras podem ser seriamente afetadas devido uma alteração vocal, como profissionais do canto e artes cênicas; nível II: profissional da voz falada para o qual uma alteração vocal de grau moderado poderia causar impacto profissional, como ocorre com a maioria dos professores, advogados, fonoaudiólogos, teleoperadores, etc.; nível III: usuário não profissional da voz que teria suas funções profissionais prejudicadas somente por uma disfonia de grau acentuado, como médicos e vendedores; nível IV: usuário não profissional não-vocal que são os indivíduos que não sofrem limitações mesmo em condições extremas de comprometimento vocal, como desenhistas, programadores de computação, escriturários, etc.. Para este estudo, optou-se pela inclusão do nível V para os participantes desempregados, aposentados e estudantes, que não entram nas classificações anteriores, conforme também utilizado outros estudos^(21,22)^.

A ESV traz a identificação de sintomas vocais e aerodigestivos. Cada questão é pontuada de 0 a 4, sendo 0 para nunca, 1 raramente, 2 às vezes, 3 quase sempre, e 4 sempre. O escore total da ESV indica o nível geral da alteração de voz (máximo 120), com subescalas de limitação da voz (máximo 60), emocional da voz (máximo 32) e físico da voz (máximo 28). O valor de corte atribuído ao escore total é de 16 pontos^(18,23)^.

O Questionário de Saúde e Higiene Vocal - QSHV foi desenvolvido e validado em português Brasileiro e fornece informações sobre o conhecimento em saúde e higiene vocal. Cada questão é assinalada de acordo com a crença do indivíduo, as respostas estão divididas em itens positivos, neutros e negativos, sendo contabilizado um ponto para cada resposta correta de acordo com o gabarito do questionário, cuja nota de corte é de 23 pontos^(19)^.

O Índice de Desvantagem para o Canto Moderno - IDCM é composto por 30 itens para explorar uma percepção de desvantagem no canto, distribuídos em três subescalas: incapacidade, desvantagem e defeito, que correspondem respectivamente aos domínios funcional, emocional e orgânico. As respostas são pontuadas de acordo com a frequência de ocorrência: 0: nunca, 1: quase nunca, 2: às vezes, 3: quase sempre e 4: sempre. Cada subescala tem valor máximo de 40 pontos e o total, composto pela somatória dos anteriores, com um escore máximo de 120 pontos. Quanto maior a pontuação, maior a desvantagem vocal no canto percebida pelo indivíduo Este protocolo tem seu original em italiano e foi traduzido e adaptado culturalmente ao português brasileiro^(15)^.

### Análise de dados

Os dados foram analisados de forma descritiva e inferencial utilizando-se o software SPSS 25.0. Foi considerado um nível de significância de 5% para as análises inferências.

Na análise descritiva das variáveis quantitativas contínuas e qualitativas ordinais foram calculadas as medidas de tendência central (média e mediana), variabilidade (desvio-padrão) e posição (mínimo, máximo, primeiro e terceiro quartis). Na análise descritiva das variáveis qualitativas nominais foram calculadas a frequência absoluta e a frequência relativa percentual.

As variáveis quantitativas foram analisadas com o Teste Shapiro-Wilk e todas apresentaram distribuição não-normal. A análise inferencial de comparação de variáveis quantitativas e qualitativas ordinais em função de dois grupos independentes foi realizada com o Teste de Mann-Whitney. A análise inferencial de comparação de variáveis quantitativas e qualitativas ordinais em função de múltiplos grupos independentes foi realizada com o Teste Kruskal-Wallis. Os valores significativos foram submetidos a análise por Pairwise. A correlação entre variáveis qualitativas ordinais e quantitativas foi realizada com o Teste de Correlação de Spearman. Para a análise da força das correlações foi usado o coeficiente Kappa de Landis e Koch^(24)^.

## RESULTADOS

Participaram do presente estudo 112 líderes religiosos do Candomblé no Brasil, sendo a média de idade de 42,63 anos, variando de 17 a 67 anos e com média de tempo de iniciado na religião de 18,93 anos, variando de menos de um ano a 52 anos. A análise descritiva de caracterização da amostra pode ser encontrada na Tabela 1.

**Tabela 1:** Análise descritiva das variáveis qualitativas nominais de caracterização da amostra em líderes religiosos do Candomblé no Brasil

Variável e categorias	N	%
Raça / cor		
Branco	20	17,86
Preto	70	62,50
Pardo	21	18,75
Outros	1	0,89
Gênero		
Masculino	56	50,00
Feminino	56	50,00
Categorização da profissão		
I	9	8,04
II	30	26,79
III	22	19,64
IV	39	34,82
V	12	10,71
Cargo religioso no Candomblé		
Ogan	20	17,86
Liderança Pai	28	25,00
Liderança Mãe	35	31,25
Egbomi	15	13,39
Ekede	14	12,50
Nação no Candomblé		
Ketu	85	75,89
Jeje	8	7,14
Angola	7	6,25
Nagô Egbá	3	2,68
Efon	2	1,79
Yoruba	3	2,68
Outros	4	3,57
Canta nos ritos religiosos		
Sim	112	100,00
Consultou médico otorrinolaringologista por problemas de voz no último ano/motivo		
Não	100	89,29
Dor de garganta	6	5,36
Rouquidão	3	2,68
Perda da voz	1	0,89
Outros	2	1,79
Terapia fonoaudiológica		
Não	93	83,04
Sim	15	13,39
Outros	4	3,57

Análise descritiva

**Legenda:** N = frequência absoluta; % = frequência relativa

Como pode ser observado na Tabela 1, na distribuição do quesito raça \ cor houve uma maioria de sujeitos que se autodeclaram pretos (62,50%). Podemos notar ainda que houve distribuição igualitária entre os gêneros (50%). Quando nos debruçamos sobre a distribuição da categorização das profissões, podemos identificar que houve maior quantidade de lideranças religiosas que exercem profissões de nível IV (34,82%) seguidos de nível II (26,79%) e III (19,64%), sendo os níveis V (10,71%) e I (8,04%) foram apontados pelo menor número de sujeitos. Um outro aspecto que chama atenção é o fato de que 89,29% dos sujeitos não consultaram um médico Otorrinolaringologista no último ano, assim como 83,04% nunca fez terapia fonoaudiológica.

Visualiza-se na Tabela 2 a descrição das variáveis de autopercepção atual da voz, autopercepção de sintomas vocais, conhecimento em saúde e higiene vocal e autopercepção de desvantagem vocal no canto moderno. No que tange à autoavaliação vocal no momento atual, a média dos indivíduos está entre relato de voz boa e razoável. O ESV apontou que a média dos sujeitos apresentou escore total de 23,04 pontos, acima da nota de corte de 16 pontos do protocolo validado para o Português Brasileiro que separa indivíduos disfônicos dos vocalmente saudáveis^(18,23)^.

**Tabela 2:** Análise descritiva das variáveis quantitativas de autoavaliação em líderes religiosos do Candomblé no Brasil

Variáveis	Média	DP	Mínimo	Máximo	1Q	Mediana	3Q
Autopercepção atual da voz	2,22	0,97	0,00	4,00	2,00	2,00	3,00
ESV							
Limitação	13,15	10,86	0,00	55,00	5,00	10,00	18,00
Emocional	3,20	5,55	0,00	24,00	0,00	0,00	4,00
Físico	6,70	5,58	0,00	25,00	3,00	5,50	9,00
Total	23,04	20,21	0,00	99,00	10,00	16,00	28,75
QSHV Total	23,54	6,76	5,00	31,00	20,00	26,00	29,00
IDCM							
Incapacidade	8,80	8,10	0,00	40,00	2,00	6,50	13,00
Desvantagem	4,92	6,88	0,00	40,00	0,00	2,00	7,00
Defeito	11,94	10,01	0,00	40,00	3,00	10,00	19,00
Total	25,66	22,71	0,00	119,00	6,00	22,00	35,00

Análise descritiva

**Legenda:** DP = desvio padrão; 1Q = primeiro quartil; 3Q = terceiro quartil; ESV = Escala de Sintomas Vocais; QSHV = Questionário de Saúde e Higiene Vocal; IDCM = Índice de Desvantagem para o Canto Moderno

Com relação ao QSHV, a média do escore total foi de 23,54 pontos, dentro da normalidade do valor de corte de indivíduos saudáveis do instrumento (≥ 23 pontos)^(19)^. Já o IDCM apresentou média do escore total de 25,66; escore Incapacidade de 8,80 pontos, Desvantagem de 4,92 pontos e Defeito de 11,94 pontos, como pode ser encontrado na Tabela 2.

Houve correlação^(24)^ positiva entre a autoavaliação vocal no momento atual, todos os domínios de autopercepção de sintomas vocais e todos os domínios de autopercepção de desvantagem vocal no canto moderno em líderes religiosos do Candomblé no Brasil. Apenas o conhecimento sobre saúde e higiene vocal não se correlacionou^(24)^ com as variáveis de autoavaliação vocal no momento atual.

A autopercepção da voz evidenciou correlação positiva moderada^(24)^ com os escores ESV Limitação, ESV Emocional, ESV total IDCM Incapacidade, IDCM Defeito e IDCM Total e correlação fraca com ESV Físico e IDCM Desvantagem; O ESV Limitação apresentou correlação positiva quase perfeita^(24)^ com ESV total e correlação positiva substancial^(24)^ com ESV emocional, ESV Físico, IDCM Incapacidade, IDCM Desvantagem, IDCM Defeito e IDCM Total. O ESV Emocional evidenciou correlação positiva substancial^(24)^ com ESV Total e IDCM Desvantagem e correlação positiva moderada^(24)^ com ESV Físico, IDCM Incapacidade, IDCM Defeito, IDCM Total.

O índice ESV Físico apresentou correlação positiva quase perfeita^(24)^ com ESV Total; correlação positiva substancial^(24)^ com IDCM Defeito e IDCM Total e correlação positiva moderada^(24)^ com IDCM Incapacidade e IDCM Desvantagem.

O ESV Total evidenciou correlação positiva substancial^(24)^ com IDCM Incapacidade, IDCM Desvantagem, IDCM Defeito e IDCM Total. IDCM Incapacidade evidenciou correlação positiva quase perfeita^(24)^ com IDCM Total e substancial^(24)^ com IDCM Incapacidade e IDCM Defeito. O IDCM Desvantagem evidenciou correlação positiva substancial^(24)^ com IDCM Total e moderada^(24)^ com o IDCM Defeito. O IDCM Defeito evidenciou correlação positiva quase perfeita^(24)^ com o IDCM Total, como pode ser observado na Tabela 3.

**Tabela 3:** Correlação entre as variáveis quantitativas de autoavaliação em líderes religiosos do Candomblé no Brasil

		ESV Limitação	ESV Emocional	ESV Físico	ESV Total	QSHV Total	IDCM Incapacidade	IDCM Desvantagem	IDCM Defeito	IDCM Total
Autopercepção atual da voz	R	0,505	0,463	0,362	0,517	-0,025	0,485	0,305	0,459	0,488
	p-valor	<0,001*	<0,001*	<0,001*	<0,001*	0,791	<0,001*	<0,001*	<0,001*	<0,001*
ESV Limitação	R		0,664	0,720	0,964	0,043	0,682	0,621	0,766	0,767
	p-valor		<0,001*	<0,001*	<0,001*	0,650	<0,001*	<0,001*	<0,001*	<0,001*
ESV Emocional	R			0,511	0,736	0,038	0,577	0,628	0,504	0,604
	p-valor			<0,001*	<0,001*	0,694	<0,001*	<0,001*	<0,001*	<0,001*
ESV Físico	R				0,834	0,107	0,544	0,477	0,643	0,613
	p-valor				<0,001*	0,260	<0,001*	<0,001*	<0,001*	<0,001*
ESV Total	R					0,072	0,711	0,641	0,775	0,789
	p-valor					0,452	<0,001*	<0,001*	<0,001*	<0,001*
QSHV Total	R						0,013	-0,029	0,087	0,054
	p-valor						0,895	0,764	0,362	0,575
IDCM Incapacidade	R							0,662	0,794	0,937
	p-valor							<0,001*	<0,001*	<0,001*
IDCM Desvantagem	R								0,605	0,752
	p-valor								<0,001*	<0,001*
IDCM Defeito	R									0,929
	p-valor									<0,001*

* Valores significativos (p≤0,05) - Teste de Correlação de Spearman

**Legenda:** R = coeficiente de correlação; ESV = Escala de Sintomas Vocais; QSHV = Questionário de Saúde e Higiene Vocal; IDCM = Índice de Desvantagem para o Canto Moderno

Não houve diferença na análise das variáveis quantitativas de autoavaliação em função das variáveis cargo religioso e nação do Candomblé em líderes religiosos do Candomblé no Brasil.

Diante do fato de que não houve diferença estatística significante entre os diferentes cargos e diferentes nações da religião, passaremos a analisar o grande grupo lideranças do Candomblé do Brasil.

Houve escores significativamente maiores de autopercepção de sintomas vocais no domínio limitação (p=0,012) e total (p=0,012) e autopercepção de desvantagem vocal no canto moderno no domínio desvantagem (p=0,008) em líderes religiosos do Candomblé no Brasil do gênero feminino, em relação aos do gênero masculino, como mostra a Tabela 4.

**Tabela 4:** Análise das variáveis quantitativas de autoavaliação em função do gênero em líderes religiosos do Candomblé no Brasil

Variável	Gênero	Média	DP	Mínimo	Máximo	1Q	Mediana	3Q	p-valor
Autopercepção atual da voz	Masculino	2,13	0,95	0,00	4,00	2,00	2,00	3,00	0,330
	Feminino	2,32	0,99	0,00	4,00	2,00	2,00	3,00	
ESV									
Limitação	Masculino	11,09	10,30	0,00	45,00	4,00	8,50	14,00	0,012*
	Feminino	15,21	11,10	1,00	55,00	7,00	13,00	19,75	
Emocional	Masculino	2,91	5,74	0,00	24,00	0,00	0,00	2,75	0,137
	Feminino	3,48	5,38	0,00	22,00	0,00	1,00	4,75	
Físico	Masculino	5,73	5,22	0,00	21,00	1,25	5,00	8,00	0,052
	Feminino	7,66	5,81	0,00	25,00	3,25	6,00	10,75	
Total	Masculino	19,73	19,80	0,00	90,00	7,00	12,00	26,00	0,012*
	Feminino	26,36	20,24	2,00	99,00	12,25	22,50	31,50	
QSHV Total	Masculino	23,82	6,46	9,00	31,00	19,50	26,00	29,00	0,659
	Feminino	23,25	7,10	5,00	30,00	20,00	26,00	29,00	
IDCM									
Incapacidade	Masculino	8,02	7,65	0,00	28,00	2,00	5,50	12,75	0,233
	Feminino	9,59	8,53	0,00	40,00	4,00	7,00	13,00	
Desvantagem	Masculino	4,21	6,43	0,00	26,00	0,00	1,00	7,00	0,038*
	Feminino	5,63	7,30	0,00	40,00	1,00	3,00	7,00	
Defeito	Masculino	10,18	9,61	0,00	40,00	2,00	8,50	17,00	0,053
	Feminino	13,70	10,17	0,00	39,00	6,25	12,50	20,00	
Total	Masculino	22,41	21,47	0,00	89,00	5,00	16,00	31,75	0,093
	Feminino	28,91	23,62	0,00	119,00	13,00	25,50	40,00	

* Valores significativos (p≤0,05) - Teste de Mann-Whitney

**Legenda:** DP = desvio padrão; 1Q = primeiro quartil; 3Q = terceiro quartil; ESV = Escala de Sintomas Vocais; QSHV = Questionário de Saúde e Higiene Vocal; IDCM = Índice de Desvantagem para o Canto Moderno

Não houve correlação entre as variáveis quantitativas de autoavaliação e as variáveis idade e tempo de iniciado em líderes religiosos do Candomblé no Brasil. Houve diferença no conhecimento em saúde e higiene vocal em função da categorização da profissão em líderes religiosos do Candomblé no Brasil (p=0,008). A análise por Pairwise mostrou que profissionais do nível I apresentaram escores significativamente maiores que os profissionais do grupo IV (p=0,010) e os do nível V (p=0,008), como pode ser observado na Tabela 5.

**Tabela 5:** Análise das variáveis quantitativas de autoavaliação em função da variável categorização da profissão em líderes religiosos do Candomblé no Brasil

Variável	Categorização da profissão	Média	DP	Mínimo	Máximo	1Q	Mediana	3Q	p-valor	Pairwise
Autopercepção atual da voz	I	2,22	1,39	0,00	4,00	1,00	2,00	3,50	0,860	
	II	2,33	0,88	0,00	4,00	2,00	2,00	3,00		
	III	2,09	0,81	1,00	4,00	1,75	2,00	3,00		
	IV	2,26	0,99	0,00	4,00	2,00	2,00	3,00		
	V	2,08	1,16	0,00	4,00	1,00	2,00	3,00		
ESV										
Limitação	I	12,78	10,63	2,00	36,00	5,50	9,00	18,50	0,874	
	II	11,77	8,08	1,00	32,00	5,00	11,00	16,50		
	III	11,64	8,74	0,00	35,00	4,00	11,00	17,25		
	IV	15,74	13,68	0,00	55,00	4,00	10,00	23,00		
	V	11,25	10,25	2,00	34,00	5,00	7,00	12,75		
Emocional	I	5,11	6,43	0,00	19,00	0,50	2,00	9,00	0,410	
	II	2,53	4,76	0,00	22,00	0,00	0,00	3,25		
	III	3,09	4,40	0,00	16,00	0,00	0,50	6,00		
	IV	3,56	6,47	0,00	24,00	0,00	0,00	4,00		
	V	2,42	5,81	0,00	20,00	0,00	0,00	1,75		
Físico	I	6,78	7,50	1,00	25,00	2,00	3,00	9,00	0,554	
	II	6,53	3,79	0,00	13,00	4,00	6,00	10,00		
	III	5,82	5,07	0,00	20,00	1,00	5,00	8,25		
	IV	7,90	6,79	0,00	22,00	2,00	6,00	13,00		
	V	4,75	4,05	0,00	15,00	2,25	3,50	6,00		
Total	I	24,67	24,17	5,00	80,00	8,50	13,00	36,50	0,870	
	II	20,83	14,00	1,00	51,00	10,75	16,00	28,50		
	III	20,55	16,10	0,00	71,00	8,50	19,50	26,25		
	IV	27,21	25,59	0,00	99,00	10,00	16,00	42,00		
	V	18,42	17,57	4,00	63,00	6,75	12,00	25,25		
QSHV total	I	28,78	1,92	24,00	30,00	28,50	29,00	30,00	0,008*	I > IV (p=0,010) = V (p=0,008)
	II	24,57	6,22	8,00	31,00	24,50	26,50	29,00		
	III	24,27	4,91	12,00	30,00	21,25	25,50	28,00		
	IV	22,31	7,42	5,00	31,00	16,00	26,00	28,00		
	V	19,67	8,54	6,00	29,00	10,75	20,50	29,00		
IDCM										
Incapacidade	I	7,78	7,31	1,00	19,00	1,00	6,00	15,00	0,946	
	II	7,97	6,96	0,00	23,00	3,75	5,50	11,25		
	III	8,00	7,42	0,00	2,00	3,00	0,00	0,00		
	IV	9,97	9,32	0,00	40,00	2,00	7,00	15,00		
	V	9,33	9,05	0,00	28,00	1,50	6,50	16,75		
Desvantagem	I	6,11	6,62	0,00	17,00	0,50	3,00	13,00	0,847	
	II	4,03	6,18	0,00	26,00	0,00	2,00	4,00		
	III	3,82	4,54	0,00	16,00	0,00	2,50	7,00		
	IV	6,08	8,25	0,00	40,00	0,00	3,00	8,00		
	V	4,50	7,78	0,00	28,00	0,25	2,00	6,00		
Defeito	I	11,67	8,77	1,00	24,00	3,00	10,00	20,00	0,953	
	II	12,37	9,12	0,00	40,00	6,50	11,00	19,25		
	III	10,45	9,01	0,00	37,00	2,50	10,50	14,25		
	IV	12,23	10,65	0,00	39,00	2,00	11,00	20,00		
	V	12,83	13,50	0,00	36,00	1,25	8,00	26,00		
Total	I	25,56	22,07	3,00	59,00	5,00	26,00	48,50	0,964	
	II	24,37	20,24	0,00	589,00	12,50	21,00	33,00		
	III	22,27	19,49	0,00	82,00	5,50	21,50	32,25		
	IV	28,28	25,83	0,00	119,00	6,00	24,00	45,00		
	V	26,67	26,17	1,00	90,00	5,75	18,50	44,75		

* Valores significativos (p≤0,05) - Teste de Kruskal-Wallis

**Legenda:** DP = desvio padrão; 1Q = primeiro quartil; 3Q = terceiro quartil; ESV = Escala de Sintomas Vocais; QSHV = Questionário de Saúde e Higiene Vocal; IDCM = Índice de Desvantagem para o Canto Moderno

## DISCUSSÃO

Os desafios vocais dos religiosos do Candomblé são inúmeros, pois além do uso intensivo da voz no âmbito religioso, este uso da voz no ato religioso é muitas vezes acompanhado de outras profissões também caracterizadas como profissionais da voz. Apesar disso, pode-se observar o pouco acesso aos serviços responsáveis pelo cuidado, prevenção e de reabilitação/tratamento em saúde vocal, como a Otorrinolaringologia e a Fonoaudiologia (Tabela 1).

Neste estudo, a maior parte das respostas da autoavaliação vocal está entre voz boa e razoável, que pode explicar a baixa procura por profissionais especializados, ou ainda, como visto em cantores populares^(16)^, em que pequenas alterações vocais podem fazer parte da assinatura vocal, não sendo, em alguns casos, motivos para a busca por profissionais de saúde.

A análise de auto percepção vocal é uma estratégia comumente utilizada em pesquisas sobre voz e protocolos de autoavaliação, inclusive em estudos brasileiros para a validação de protocolos de autoavaliação em voz^(25)^ e tem se mostrado uma importante medida para o diagnóstico vocal de pacientes com disfonia^(26)^, uma vez que um problema de voz pode comprometer a qualidade de vida.

Os resultados apontam que esta população apresenta escores altos de sintomas vocais e aerodigestivos assim como a percepção do impacto e desvantagem para o canto moderno nos escores de incapacidade, desvantagem e defeito.

A média do escore do ESV-total encontrada no grupo de sujeitos foi 23,04 (Tabela 2), aproximadamente sete pontos acima do valor de corte atribuído ao escore total que separa indivíduos disfônicos dos vocalmente saudáveis (16 pontos)^(18,23)^, evidenciando que as lideranças religiosas de Candomblé estudadas apresentam e percebem sinais e sintomas de alterações vocais e aerodigestivos. Apesar disso, tal valor é menor que a média encontrada em solistas amadores da igreja batista (30,9 pontos)^(27)^. Queixas relacionadas a saúde vocal foram também encontradas na maioria dos líderes evangélicos^(5,7)^.

A média dos escores apresentados no QSHV (Tabela 2) mostra-se próxima à nota de corte, que marca a diferenciação entre o conhecimento em saúde vocal em indivíduos vocalmente saudáveis e indivíduos disfônicos. Este resultado, porém, está abaixo da média dos escores apresentados na literatura por indivíduos vocalmente saudáveis, que é 29,12 pontos^(19)^ e difere do encontrado em pastores evangélicos^(6)^ na qual a maioria dos sujeitos demonstrou ter bom conhecimento sobre saúde e higiene da voz, com escores médios de 28 pontos.

Ainda na análise descritiva, encontramos valores médios de 25,66 para o escore total do IDCM (Tabela 2). Embora não se tenha um valor de corte para o português brasileiro para este protocolo na literatura, os valores encontrados foram próximos aos encontrados em outros estudos brasileiros, como Moreti et al.^(15)^, em cantores amadores de corais com queixas vocais, com 26,91 pontos. Já em Sales et al.^(28)^, a média do escore total em cantores populares foi de 16,6 pontos, indicando menor desvantagem em relação a população encontrada neste estudo.

Neste estudo houve correlação positiva entre a autoavaliação no momento atual vocal e todos os domínios de autopercepção de sintomas vocais e todos os domínios de autopercepção de desvantagem vocal no canto moderno em líderes religiosos do Candomblé no Brasil, que pode evidenciar que quanto mais o indivíduo percebe sua voz alterada, menor seu rendimento vocal (Tabela 3). Um problema vocal pode refletir em como o sujeito avalia sua própria voz e, embora nem todas as disfonias se manifestem por alteração na qualidade vocal, a literatura aponta uma correlação positiva entre a auto percepção do problema vocal e o impacto negativo na qualidade de vida^(25,26)^.

Houve correlação estatisticamente significativa entre o conhecimento sobre saúde e higiene vocal e a variável de autoavaliação vocal no momento atual (Tabela 3), resultado diferente do encontrado por Coelho et al.^(16)^, em que a percepção de sintomas vocais não teve relação com o grau de conhecimento de saúde e higiene vocal nos cantores estudados e diferente de Roza et al.^(17)^, em que indivíduos com mais conhecimento em cuidados vocais evidenciaram melhor autoavaliação de voz. Assim, como os pastores, as lideranças do Candomblé podem ser consideradas parte de uma população com risco vocal no ambiente laboral^(6)^.

A correlação positiva entre a autoavaliação vocal e ESV e IDCM (Tabela 3) evidencia que quanto maior o escore de sintomas referidos, maior é a desvantagem do sujeito com relação ao canto, como também demonstrado por Lopes e Ghirard^(27)^.

Apenas o conhecimento sobre saúde e higiene vocal não se correlacionou com as variáveis de autoavaliação vocal (Tabela 3), apontando que o conhecimento em saúde vocal, pode não ser suficiente para garantir a redução de sintomas vocais e aerodigestivos.

Não houve correlação entre as variáveis quantitativas de autoavaliação e as variáveis idade e tempo de iniciado em líderes religiosos do Candomblé no Brasil (Tabela 3), diferente do estudo realizado por Lobo et al.^(6)^ que encontrou em pastores com maior tempo de carreira pastoral escores mais elevados no QSHV.

Houve escores significativamente maiores de autopercepção de sintomas vocais no domínio limitação e total da ESV e autopercepção de desvantagem vocal no canto moderno no domínio desvantagem no gênero feminino, em relação aos do gênero masculino (Tabela 4).

Essa relação de escores maiores no gênero feminino foi encontrada também em cantoras evangélicas e entre cantores e cantoras do erudito e do popular^(5,8,16)^. Para estes pesquisadores, o fato de as mulheres apresentarem maior desvantagem vocal do que os homens pode indicar a capacidade de perceberem com maior clareza qualquer restrição na habilidade de exercer o canto, seja em questões de natureza orgânica, funcional ou emocional^(8)^, diferente de Souza et al.^(7)^ que encontrou prevalência no sexo masculino.

Como pudemos observar, o conhecimento em saúde e higiene vocal pode não ter sido suficiente para a não percepção de sintomas vocais, dado que esta variável não se correlacionou estatisticamente com as demais.

Sobre a categorização da profissão em líderes religiosos (Tabela 5), identificamos no QSHV que profissionais do nível I (elite vocal) apresentaram escores significativamente maiores que os profissionais do grupo IV (indivíduos que não sofrem limitações mesmo em condições extremas de comprometimento vocal e os aposentados). Este resultado pode sugerir que o conhecimento em saúde e higiene vocal está mais relacionado a categoria profissional que à função religiosa. Importante destacar a relação entre trabalho e distúrbios vocais, uma vez que trabalhadores que usavam a voz profissionalmente apresentaram maior frequência de distúrbios vocais relacionados ao trabalho, o que evidencia cada vez mais que ações referentes aos cuidados com o uso da voz para profissionais que a usam como instrumento de trabalho, entre elas as lideranças religiosas, tornam-se cada vez mais relevantes^(29,30)^.

Assim, a fonoaudiologia pode contribuir, dentro das instituições religiosas, atuando na prevenção de possíveis alterações vocais, já que os sujeitos possuem pouco conhecimento sobre o uso de voz^(1)^.

Considerando a natureza de coleta via formulário online, variáveis como a autoavaliação vocal no momento atual, que evidenciam como o olhar do sujeito sobre a qualidade de sua voz, ganham uma maior importância.

Os protocolos utilizados nesta pesquisa foram complementares, visto que contemplam dimensões diferentes da autopercepção vocal, quantitativa e qualitativa, conhecimento em saúde e higiene vocal e a percepção da desvantagem para o canto, na tentativa de envolver o indivíduo em sua integralidade.

A partir deste estudo, podemos afirmar que o conhecimento sobre saúde e higiene vocal apresentado pelas lideranças religiosas do Candomblé do Brasil atua como um redutor de risco para o desenvolvimento de alterações na voz, mas este conhecimento precisa ser aplicado na rotina diária, visto que, isoladamente, não garante a saúde vocal^(6)^.

Consideramos importante pensar que juntamente com a autopercepção, a aplicação de protocolos de avaliação da voz pode trazer informações mais completas sobre a qualidade vocal desta população.

Vale ressaltar que existe um número reduzido de pesquisas sobre o canto nas religiões de matriz africanas, como a Umbanda e o Candomblé, sendo este um campo importante para pesquisas e trabalhos dentro da Fonoaudiologia, dados os escores aumentados tanto no protocolo relacionado à sintomas vocais e aerodigestivos quanto na desvantagem no canto. Destaca-se ainda a necessidade de ampliação de ações de cuidado em saúde e higiene vocal, pois a maioria dos líderes tende a praticar o canto e a função religiosa durante anos sem nunca ter estudado o assunto ou fazer uso de técnicas vocais^(8)^.

## CONCLUSÃO

As lideranças de Candomblé brasileiro estudadas evidenciaram percepção de sinais e sintomas de alterações vocais e aerodigestivas, assim como desvantagem vocal para o canto. Tal percepção foi mais evidente nas mulheres.

Os diferentes líderes religiosos apresentaram bom conhecimento de saúde vocal independente do gênero. No entanto, este conhecimento não teve correlação com os sintomas vocais autopercebidos nem com as desvantagens vocais vivenciadas para o canto.

Apesar da identificação dos sintomas vocais, a maior parte dos sujeitos relatou não ter acessado profissionais de saúde responsáveis por esses cuidados com a voz no último ano.

Há a percepção de que tais sintomas influenciam na autoavaliação da voz, porém pode-se perceber que somente o conhecimento em saúde vocal, não foi suficiente para a proteção desta população. É muito importante a sensibilização da mesma e promoção de acesso a serviços de saúde vocal.
